# Changes in overall ventricular myocardial architecture in the setting of a porcine animal model of right ventricular dilation

**DOI:** 10.1186/s12968-017-0404-0

**Published:** 2017-11-27

**Authors:** Peter Agger, Christine Ilkjær, Christoffer Laustsen, Morten Smerup, Jesper R. Frandsen, Steffen Ringgaard, Michael Pedersen, John B. Partridge, Robert H. Anderson, Vibeke Hjortdal

**Affiliations:** 1 Department of Cardiothoracic & Vascular Surgery, Aarhus University Hospital, Skejby, Aarhus, Denmark; 2 Center for Functionally Integrative Neuroscience, Aarhus University Hospital, Aarhus, Denmark; 3 MR Research Center, Aarhus University Hospital, Aarhus, Denmark; 4 Institute of Genetic Medicine, Newcastle University, Newcastle-upon-Tyne, UK; 5 Comparative Medicine Lab, Aarhus University Hospital, Aarhus, Denmark; 6 Department of Clinical Medicine, Aarhus University, Aarhus, Denmark; 7 Department of Cardiothoracic Surgery, Rigshospitalet, Copenhagen, Denmark; 8 Eurobodalla Unit, Rural Clinical School of the ANU College of Medicine, Biology & Environment, Batemans Bay, NSW Australia

**Keywords:** Myocardial remodeling, Diffusion tensor imaging, Heart failure, Congenital heart disease

## Abstract

**Background:**

Chronic pulmonary regurgitation often leads to myocardial dysfunction and heart failure. It is not fully known why secondary hypertrophy cannot fully protect against the increase in wall stress brought about by the increased end-diastolic volume in ventricular dilation. It has been assumed that mural architecture is not deranged in this situation, but we hypothesised that there might be a change in the pattern of orientation of the aggregations of cardiomyocytes, which would contribute to contractile impairment.

**Methods:**

We created pulmonary valvular regurgitation by open chest, surgical suturing of its leaflets in seven piglets, performing sham operations in seven control animals. Using cardiovascular magnetic resonance imaging after 12 weeks of recovery, we demonstrated significantly increased right ventricular volumes in the test group. After sacrifice, diffusion tensor imaging of their hearts permitted measurement of the orientation of the cardiomyocytes.

**Results:**

The helical angles in the right ventricle approached a more circumferential orientation in the setting of right ventricular RV dilation (*p* = 0.007), with an increased proportion of surface-parallel cardiomyocytes. In contrast, this proportion decreased in the left ventricle. Also in the left ventricle a higher proportion of E3 angles with a value around zero was found, and conversely a lower proportion of angles was found with a numerical higher value. In the dilated right ventricle the proportion of E3 angles around −90° is increased, while the proportion around 90° is decreased.

**Conclusion:**

Contrary to traditional views, there is a change in the orientation of both the left ventricular and right ventricular cardiomyocytes subsequent to right ventricular dilation. This will change their direction of contraction and hinder the achievement of normalisation of cardiomyocytic strain, affecting overall contractility. We suggest that the aetiology of the cardiac failure induced by right vetricular dilation may be partly explained by morphological changes in the myocardium itself.

## Background

Right ventricular dilation is often caused by pulmonary valve regurgitation, which is predominantly seen in congenital heart disease following balloon dilation of critical pulmonary stenosis, or perforation of valvar pulmonary atresia [1]. The most important, and well-described, clinical context for pulmonary regurgitation, however, is in patients with repaired tetralogy of Fallot, the most common cyanotic congenital heart disease [2].

**Fig. 1:**
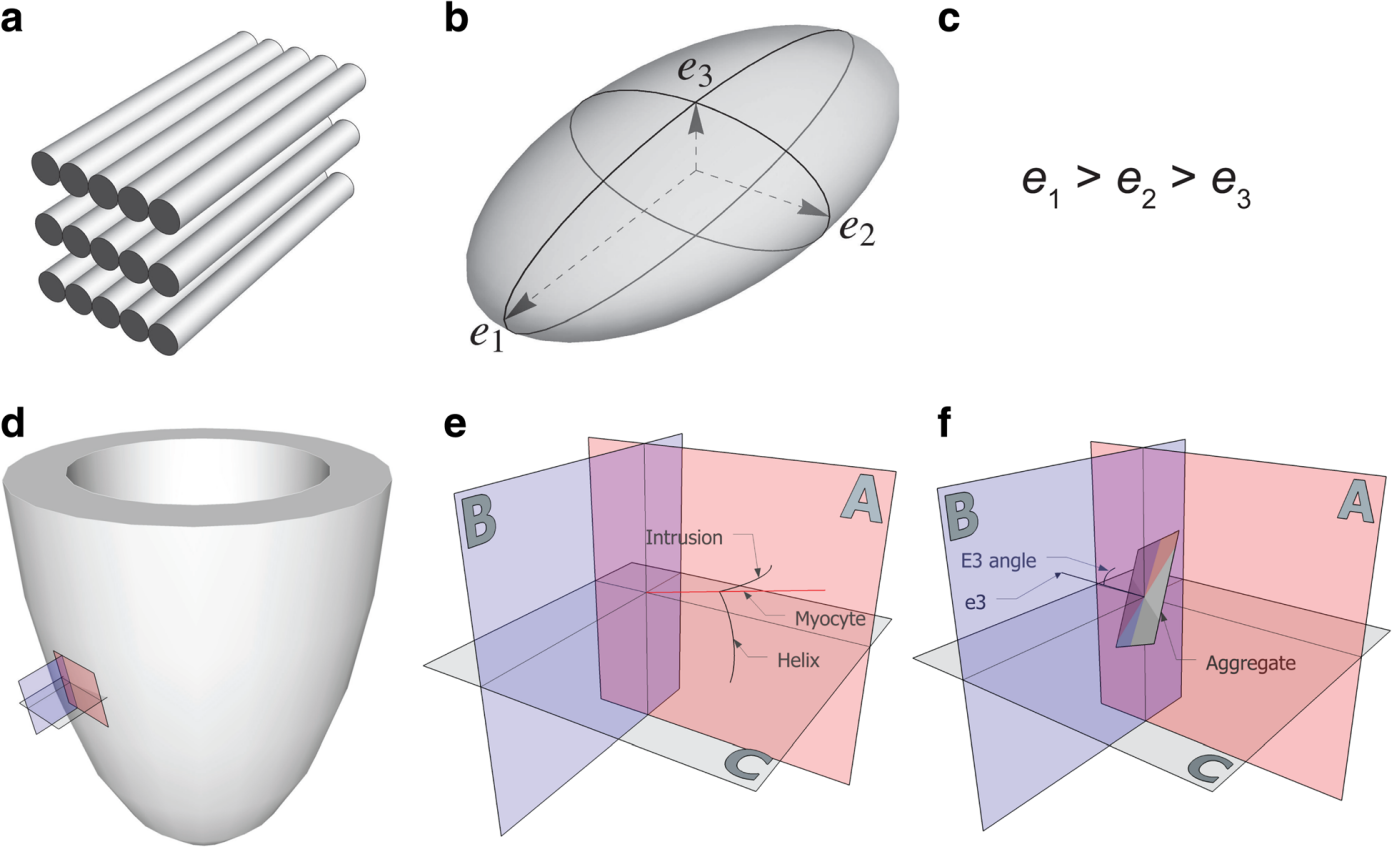
Diffusion tensor and eigenvectors. The shape of the diffusion tensor in an ordered fibrous environment such as the myocardium. Panel **a** depicts the schematic ordering of the cardiomyocytes while panel **b** shows the coherent shape of the diffusion tensor, the primary (e1), secondary (e2) and tertiary (e3) eigenvectors are shown along with their relationship in panel **c**. Panel **d** shows a schematic representation of the left ventricle with the local orthogonal planes aligned relative to the epicardium. Panel **e** shows the local set of orthogonal planes as superimposed in every voxel. Plane A (red) is the circumferential-longitudinal plane parallel to the epicardial tangential plane and plane B (blue) is the radial-longitudinal plane parallel to the left ventricular long axis and orthogonal to plane A. Plane C, the local “horizontal” plane, is the circumferential-radial plane orthogonal to planes A and B. The helical angle is the angle between the cardiomyocyte and plane C. The intrusion angle is the angle between the cardiomyocyte and plane A. Panel **f** shows the E3 angle as measured using the tertiary eigenvector (e3) relative to plane B. The aggregation of cardiomyocytes is depicted as a flat square, which is a gross oversimplification

Even though the treatment of tetralogy of Fallot has improved dramatically in recent times, serious complications, like right ventricular dilation as a result of longstanding pulmonary regurgitation, still develop as an almost unavoidable result of the treatment [3, 4]. These results in an increased risk of heart failure, severe arrhythmias, and sudden death [1, 2, 4]. Dilation is a natural consequence of right ventricular volume overload. The ventricles have to dilate in order to accommodate the increased blood load because the two other possible responses, namely decreased end-systolic volume or increased heart rate, are more difficult to achieve. As of today, it is not known why the right ventricle, in the long term, so poorly tolerates dilation, nor why this ultimately will result in heart failure. Rearrangement of the cardiomyocytes making up the ventricular walls has been shown in various cardiac diseases [5–7] and may very well be a contributing cause of heart failure in the dilated right heart. To achieve better understanding of heart failure as a consequence of right ventricular dilation, it is axiomatic that further knowledge of the myocardial architecture of the right ventricle is needed in the setting of these pathological conditions. Previously, it has been manual dissection and histological examination of pieces of the myocardium that have been the methods of choice when assessing mural architecture [8–10]. As we have pointed towards earlier, the major drawback of these approaches is that they fail to assess the microstructure of the myocardium as a three-dimensional entirety. Diffusion tensor cardiovascular magnetic resonance now provides the means to circumvent this problem, even though, as yet, the technique is incapable of visualising the individual cardiomyocytes. It has been extensively used to characterise myocardial architecture in autopsied [11] and beating hearts [12], in both health [13] and disease [6], and has been used in a variety of species. The technique uses the spontaneous self-diffusion of water as a surrogate measure of the average long-axis orientation of cardiomyocytes within defined portions of the myocardium contained within a voxel. The diffusion pattern within each voxel is described as a mathematical construct called a tensor. A tensor is defined by its three orthogonal eigenvectors [14]. It is generally accepted that the primary eigenvector of the diffusion tensor follows the orientation of the long axis of the aggregated cardiomyocytes (Fig. 1). When assessing the orientation of the tertiary eigenvector, it is found to change its orientation through the cardiac cycle [15]. In disease states, furthermore, this change is associated with ventricular thinning or thickening [6]. It has been suggested that the tertiary eigenvector reflects the orientation of anatomical substructures of cardiomyocytes that are mainly of a flattened nature, but otherwise of unknown extent and appearance. These substructures have been referred to as lamellar units [16, 17], sheets [18], or sheetlets [5]. All these names have advantages and disadvantages. As of today no study has provided a complete anatomical description of these substructures. It has been stated that they are approximately four to six cardiomyocytes in thickness [19], which is probably a gross oversimplification according to more recent studies [17], but the extent and size of the structures are still unknown. It is, therefore, difficult to assess the orientation of such poorly defined structures, and even more difficult to assign a proper name for them. In this light, in spite of having done so in previous contributions, we now believe it is preferable to refrain from characterising the aggregations by name at present.

We have previously investigated the alterations in the right ventricular myocardial architecture in two animal models of increased right ventricular pressure overload [6, 20]. We were able to show that the myocardial architecture does change as a consequence of increased afterload brought upon by persistent pulmonary hypertension in the newborn [6]. Since dilation arises from a different pathogenetic mechanism as compared to hypertophy, this raised the question as to whether such morphological changes are also to be found in the setting of right ventricular dilation. For this purpose, we have introduced a porcine model of right ventricular dilation [21] and heart failure [22]. In this study, we aimed to compare the orientations of the aggregated cardiomyocytes in the normal and dilated right ventricular myocardium.

## Methods

### Animal experiments

Fourteen female 5 kg Danish landrace pigs were studied. The animals were randomised into two groups of equal size (*N* = 7). Each animal was pre-anaesthetised with midazolam (0.5 mg/kg) and azaparone (0.5 mg/kg). Intravenous access was established through an ear vein. The pre-anaesthesia was supplemented intravenously with propofol (3 mg/kg) to allow endotracheal intubation and coupling to a ventilator. Anaesthesia was maintained by 3% inhalational sevoflurane, and analgesia was achieved with fentanyl (25 μg/kg/h) before surgery. Postoperative analgesia was achieved with flunixine (25 mg). Antibiotics consisted of penicillin, given as a dose of 100,000 IU before surgery. Neuromuscular block was obtained using pancuronium at a dose of 0.2 mg/kg at the beginning of surgery. In the group destined for right ventricular dilation, having obtained assess through a left lateral thoracotomy, we exposed the pulmonary trunk. We placed 4 to 6 sutures through its wall to secure the valvar leaflets to the inside of the root, thus creating the substrate for pulmonary valve regurgitation. Having evacuated the pneumothorax, we closed the incision in three layers and aroused the piglet, confirming the success of the procedure by postoperative echocardiography.

### Follow-up examinations

After 12 weeks, we again anaesthetised the animals using the same protocol as outlined above. After conventional cardiovascular magnetic resonance scanning the animals were brought to the experimental operating theatre, where we removed the heart through a median sternotomy, having administered 10,000 IU of heparin. Subsequent to excision, we infused 1 l of potassium rich cold cardioplegic solution (Kardioplex; H/S Apoteket, Copenhagen, Denmark) directly through the coronary arterial orifices at a pressure of approximately 100 mmHg at the point of the tip of the catheter. In order to maintain the normal end-diastolic state, we then injected a thin slurry of water-based MRI compatible polymer into the ventricles via the orifices of the atrioventricular valves. To avoid excess ventricular dilation, we injected the polymer until it escaped smoothly via the pulmonary and aortic valvar orifices. Having given approximately 15 min for the polymer to harden, the hearts were perfused with formalin at pH 7.4 using the same method as with the cardioplegic solution outlined above. The hearts were then stored submerged in formalin for a minimum of 24 h, perfused with phosphate buffered solution, also at pH 7.4, and stored at 4–5 °C until scanning.

### Imaging sequences

#### Cardiovascular magnetic resonance imaging

Cardiovascular magnetic resonance was performed with a 3.0 T system (Siemens Skyra; Siemens Healthcare, Erlangen, Germany). For the initial scans, the piglets were again anaesthetised, mechanically ventilated, and placed on the scanner bed in supine position. The orientation of the left ventricular long axis was determined using scout images. A stack of 12 contiguous short-axis slices encompassing the ventricles from base to apex was acquired during end-expiratory apnea using a retrospective, electrocardiogram -triggered balanced-steady-state-free-precession breath-hold cine sequence. Imaging parameters were set as follows: repetition time = 3.8 ms, echo time = 1.67 ms, flip angle = 43°, acquisition matrix = 336 × 235, field of view = 340 × 273 mm^2^, spatial in-plane resolution = 1.01 × 1.45 mm^2^, slice thickness = 6 mm, number of heart phases = 40.

#### Flow imaging

For measuring cardiac output, we used a phase contrast flow sequence. The measurement slice was positioned across the pulmonary trunk at the level of the sinotubular junction, using a sequence triggered by the electrocardiogram, but running during free breathing. The sequence parameters were set as follows: field of view = 200 × 200 mm^2^, acquisition matrix = 128 × 128, in-plane resolution = 1.56 × 1.56 mm^2^, slice thickness = 3.2 mm, repetition time = 15.6 ms, echo time = 4.63 ms, flip angle = 15°, flow encoding velocity = 200 cm/s. The number of cardiac frames was set to 86 and the overall scan time was 3 min.

#### Diffusion tensor cardiovascular magnetic resonance imaging

For the purposes of scanning the excised hearts, the scans were performed with an Agilent 9.4 T preclinical MRI system (Agilent, Santa Clara, CA), equipped with 400 mT gradients and vnmrJ 4.0 software. The hearts were placed with the left ventricular long-axis aligned parallel to the axis of the main magnetic field. Room temperature was maintained constant at 22.0 ± 1.5 °C and humidity at 50 ± 10%. Measurements were performed using a standard multi-slice 2D spin-echo sequence with an in-plane voxel resolution of 400 × 400 μm^2^. Repetition time: 7000 ms, echo time: 30 ms. Using 30 isotropically distributed diffusion directions [23] with the b-factor equal to 1000 s/mm^2^ and one with b = 0 s/mm^2^, 125 slices with 800 μm slice thickness were acquired. Scan time was approximately 15 h for each heart.

### Anatomical measurements

The left and right ventricular myocardial masses were subdivided into 23 zones as previously described [6]. Using the in-vivo cardiovascular magnetic resonance data, mean left ventricular wall thickness was measured by four measurements at the level of the papillary muscles in zones 7 and 10, between zones 8 and 9, and between zones 11 and 12. The left ventricular anterior to posterior ventricular diameter (AP), and septal to lateral free wall dimension (SL), were also measured, permitting calculation of the SL/AP ratio as a surrogate measure of septal deviation. We were unable to measure right ventricular mural thickness in-vivo within an acceptable error margin due to insufficient spatial resolution. Left ventricular volume through the cardiac cycle was assessed and cardiac index was calculated. Likewise, we measured right ventricular volume along with its dimensions in terms of length and distance from the septum to the free wall. The pulmonary regurgitation volume was estimated using the acquired flow data. All image analyses of in-vivo data were done using the freely available software Segment version 2.0 R4942 (http://segment.heiberg.se) [24]. Assessment of the ventricular mural diastolic thicknesses in diastole ex-vivo was achieved using the diffusion-weighted images, taking the distance between the most epicardial and the most endocardial voxel in the centre of each zone.

### Measurements of myocardial architecture

Diffusion tensor imaging data were visualised using custom made software [6, 13]. The three eigenvectors of each voxel within the myocardium were calculated. The vector data was subsequently imported in Mathematica 9 (Wolfram Research, Inc., Champaign, Illinois, USA (2012)). The datasets were rotated aligning the left ventricular long axis with the z-axis of the overall coordinate system. Three short axis slices of interest were selected, one from the middle of the basal third of the heart, one from the equatorial third being the level of the papilary muscles and lastly one from the middle of the apical third of the heart. Papillary muscles, interventricular hinge points and the apical vortex were excluded by omitting analysis of zones 1, 4, 10, 12 and 17. Conversely, endocardial trabeculations were included in the analyses because they play an important role in the myocardial contraction. We are aware that this inclusion can introduce partial volume effects especially in the transition zone between compact myocardium and trabeculations. We consider this to have only minor impact on our results owing to the high resolution of our data.

**Fig. 2:**
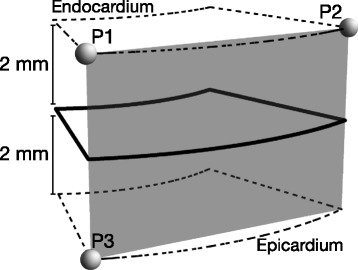
The epicardial tangential plane. Calculation of the epicardial tangential plane. The short axis circumference of the ventricle was subdivided into 64 parts of equal size as imaged. The area of interest as outlined in bold was exported with its corresponding parts 2 mm above and below as outlined in dashed lines. Three points P1, P2, and P3 were selected in the epicardium as shown and from these the epicardial tangential plane was calculated

Recognising the importance of assessing angulation of cardiomyocytes relative to the epicardial curvature, we selected the two slices 2 mm above and below each slice of interest for the purpose of calculating epicardial tangential planes as outlined in Fig. 2. A total of 64 epicardial tangential planes were calculated around the circumference of each slice of interest in the left ventricle. A similar approach was used in the right ventricle, the number of planes depending on the size of the ventricle. We then calculated the helical and intrusional angles of the cardiomyocytes along with the orientation of the aggregates of cardiomyocytes in each voxel. On average, we analysed 20,200 voxels for each heart. We defined the helical angle as the angle between the primary eigenvector and the local short axis plane [6]. The intrusional angle has, in earlier works, been defined in many different ways. We chose to use the angle between the primary eigenvector and the epicardial tangential plane. There are several current opinions, furthermore, as how correctly to assess the orientation of the aggregates of cardiomyocytes, also previously described as lamellar units, sheets or sheetlets, in particular as to whether the secondary [5] or the tertiary eigenvector [6] should be used. We assessed the orientation of the aggregated cardiomyocytes using the angle between the tertiary eigenvector (e_3_), which is the normal of the plane of the aggregate, and the epicardial tangential plane (Fig. 1). Because of the unclear nature of the aggregations and the lack of consensus regarding their name, we will describe this angle as simply the E3 angle. The E3 angle represents an average assessment of the mean orientation of the myocardial aggregates within a given voxel and is hence a measure of myocardial deformation on a cellular level.

We assessed the overall three-dimensional mural architecture using a custom-made FACT tractography algorithm [13, 25]. We selected a number of voxels, and then permitted the software to track through the primary eigenvectors, using a fractional anisotropy threshold of 0.15 and an inner product of 0.75 as previously described [26, 27]. By colour-coding the tracks, we were able to distinguish up to six different pathways. Since the total number of voxels is enormous, we selected predetermined myocardial locations for tractography. These were the right vetricular free wall at the rightmost, posterior, and the anterior aspects at the level of the left ventricular papillary muscles, and the septal right ventricular myocardium at the same level.

### Statistical analyses

Initially normality was tested in all variables on individual subject level using quantile plots, histograms and Shapiro-Wilk test. Anatomical and haemodynamic data were compared between groups using Wilcoxon ranksum test. Differences between the groups of the distributions of each angle type were tested using two-sample Kolmogorov-Smirnov test. Helical and intrusion angles were binned for each zone relative to myocardial level in 10% intervals where the overall median angle for each bin was calculated and compared between groups using Mann-Whitney U-test. Data are reported as medians with interquartile range in parentheses. Note that E3-angle data were heterogeneously distributed throughout the myocardium, thus angle differences based on myocardial level was not tested. All statistical tests presumed a significance level of 5%. Stata Statistical Software, release 11 (StataCorp LP, College Station, Texas, USA) and Mathematica 9 (Wolfram Research, Inc.) was used for statistical analyses.

## Results

### Cardiovascular magnetic resonance imaging

We examined all seven animals with pulmonary regurgitation, along with seven controls. The results of ventricular dimensions and flow are shown in Table 1. No difference between groups was found in terms of weight, body surface area, and heart rate. Likewise, no difference was found when comparing left ventricular parameters, including mural thickness and cardiac index. Right ventricular end-diastolic volume was significantly increased to 86 (70–116) ml/m^2^ in animals with pulmonary regurgitation as compared with 53 (48–76) ml/m^2^ in controls (*p* = 0.006). Similarly, Right ventricular  end-systolic volume increased to 40 (36–54) ml/m^2^ in the animals with pulmonary regurgitation versus 27 (20–40) ml/m^2^ in controls (*p* = 0.01). Regurgitation volumes were 12.6 (4.2–19.6) ml/m^2^ in the setting of pulmonary regurgitation, and 0.34 (0.12–0.62) ml/m^2^ in controls (*p* = 0.002). This gave regurgitation fractions of 39 (17–44)% versus 1.5 (0.3–2.9)% (p = 0.002). The length of the right ventricle did not change in the setting of dilation, but the width increased from 26 (19–29) mm in the controls to 29 (27–32) mm in the animals with valvar regurgitation (p = 0.01). No differences were found in post mortem ventricular mural thicknesses for either ventricle.

**Table 1:** Cardiovascular MRI assessment

	PI	Control	*P* value
Morphometric indices			
Number of animals	7	7	
Weight (kg)	21.9 (16–29)	23.7 (20–27)	NS
Body surface area (m^2^)	0.56 (0.46–0.67)	0.58 (0.52–0.63)	NS
Heart rate (bpm)	78 (57–106)	72 (57–116)	NS
Left ventricular indices			
EDV (ml/m^2^)	66 (57–81)	71 (57–82)	NS
ESV (ml/m^2^)	33 (22–38)	28 (23–46)	NS
Stroke volume (ml/m^2^)	36 (28–49)	38 (26–50)	NS
Ejection fraction (%)	53 (48–60)	57 (45–65)	NS
Cardiac index (l/min/m^2^)	2.5 (1.5–4.4)	2.6 (2.2–3.3)	NS
LV SL/AP ratio, systole	1.04 (0.89–1.15)	1.05 (1.03–1.13)	NS
LV SL/AP ratio, diastole	0.98 (0.89–1.16)	0.98 (0.91–1.03)	NS
In-vivo wall thickness, systole (mm)	10.7 (10.3–12.6)	11.6 (9.6–13.4)	NS
In-vivo wall thickness, diastole (mm)	7.4 (6.9–8)	7.4 (6.6–8.7)	NS
Ex-vivo wall thickness, diastole (mm)	6.7 (5.1–8.3)	6.6 (5.8–7.5)	NS
Right ventricular indices			
RVEDV (ml/m^2^)	86 (70–116)	53 (48–76)	0.006
RVESV (ml/m^2^)	40 (36–54)	27 (20–40)	0.01
RV ejection fraction (%)	48 (41–73)	50 (38–55)	NS
RV regurgitation volume (ml/m^2^)	12.6 (4.2–19.6)	0.34 (0.12–0.62)	0.002
RV regurgitation fraction (%)	39 (17–44)	1.5 (0.3–2.9)	0.002
Ventricular length, systole (mm)	63 (58–77)	64 (53–70)	NS
Ventricular length, diastole (mm)	75 (68–80)	75 (68–89)	NS
Septum-lateral distance, systole (mm)	21 (16–28)	18 (14–24)	NS
Septum-lateral distance, diastole (mm)	29 (27–32)	26 (19–29)	0.01
Ex-vivo wall thickness, diastole (mm)	3.7 (3.2–4.2)	3.4 (2.6–4.2)	NS

Data presented as medians (interquartile range). Medians compared using Wilcoxon Ranksum Test

*PI* pulmonary insufficiency, *NS* statistically nonsignificant, *EDV* end-diastolic volume, *ESV* end-systolic volume, *RVEDV* right ventricular end-diastolic volume, *RVESV* right ventricular end-systolic volume, *RV* right ventricle, *LV SL/AP* left ventricular septum-lateral versus anterior-posterior ratio

### Diffusion tensor imaging

**Fig. 3:**
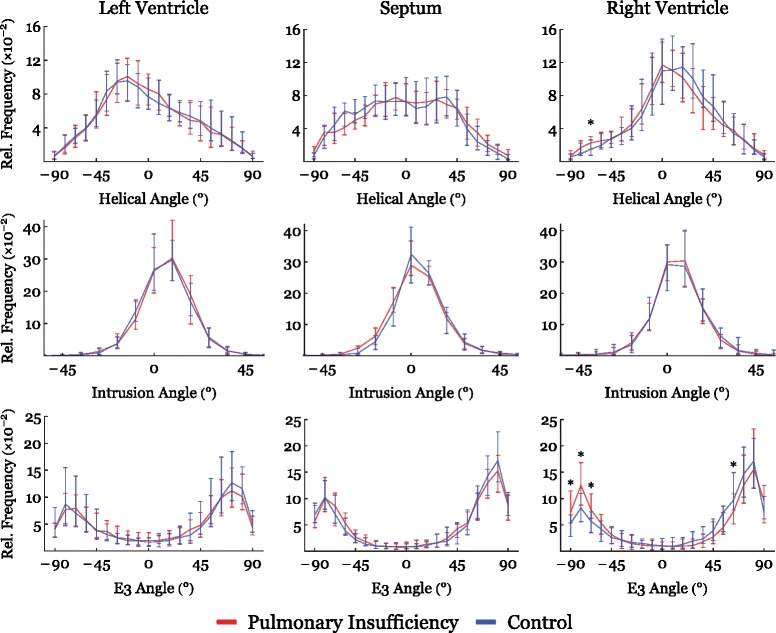
Angle histograms. Pooled results of angle calculations from the basal, equatorial and apical regions presented as histograms. Left column: Left ventricle. Middle column: Interventricular septum. Right column: Right ventricle. Top row: Results of helical angle distributions. Middle row: Results of intrusion angle distribution. Bottom row: Results of E3 angle distribution. Results are shown as medians with interquartile range. Asterisk indicates statistical significance in individual bins in post-hoc testing using Mann-Whitney U-test. Within each plot the distributions of the two groups are compared using the Kolmogorov-Smirnov test of the equality of distributions. Resulting *p*-values are as follows: Left Ventricle: Helical angle; *p* = 0.06, Intrusion angle; *p* = 0.04, E3 angle; *p* < 0.001. Septum: Helical angle; *p* = 0.71, Intrusion angle; *p* = 0.12, E3 angle; *p* = 0.56. Right Ventricle: Helical angle; *p* = 0.03, Intrusion angle; *p* = 0.007, E3 angle; *p* = 0.018

**Fig. 4:**
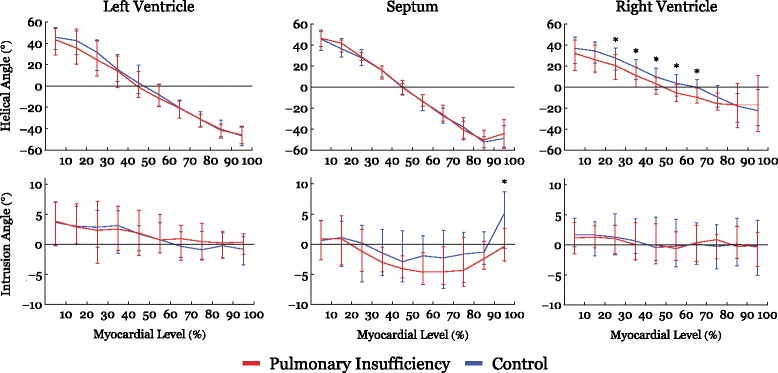
Results of helical and intrusion angles as a function of myocardial depth in percent. 0% is the sub-endocardium, 100% is the sub-epicardium. In the interventricular septum 100% is the right ventricular sub-endocardium. Results are shown as medians with interquartile range. Asterisk indicates statistical significance in individual areas in post-hoc testing using Mann-Whitney U-test

**Fig. 5:**
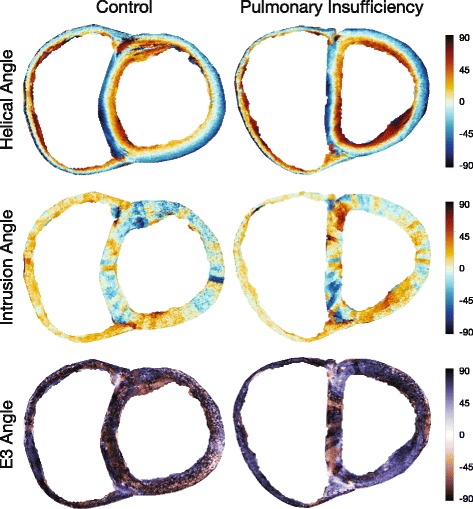
Surface plots of helical, intrusion and unit angles in control heart (left) versus heart with pulmonary regurgitation and right ventricular dilation (right). The subdivision of the myocardium into smaller parts is obvious when calculating intrusion angles since this particular angle is highly sensitive to inhomogeneities in the epicardial surface

**Fig. 6:**
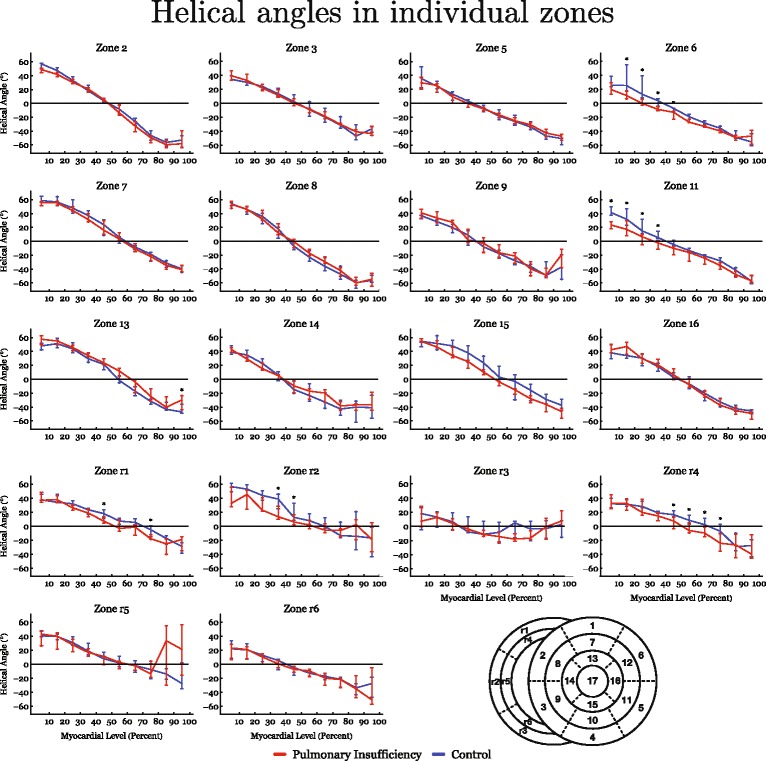
Detailed regional analysis based on data from individual zones in the entire heart. Transmural analyses of helical angles from sub-endocardium (0) to sub-epicardium (100). Asterisk indicates statistical significance in individual areas using Mann-Whitney U-test. In the down right corner a schematic representation of the zones is presented for reference

**Fig. 7:**
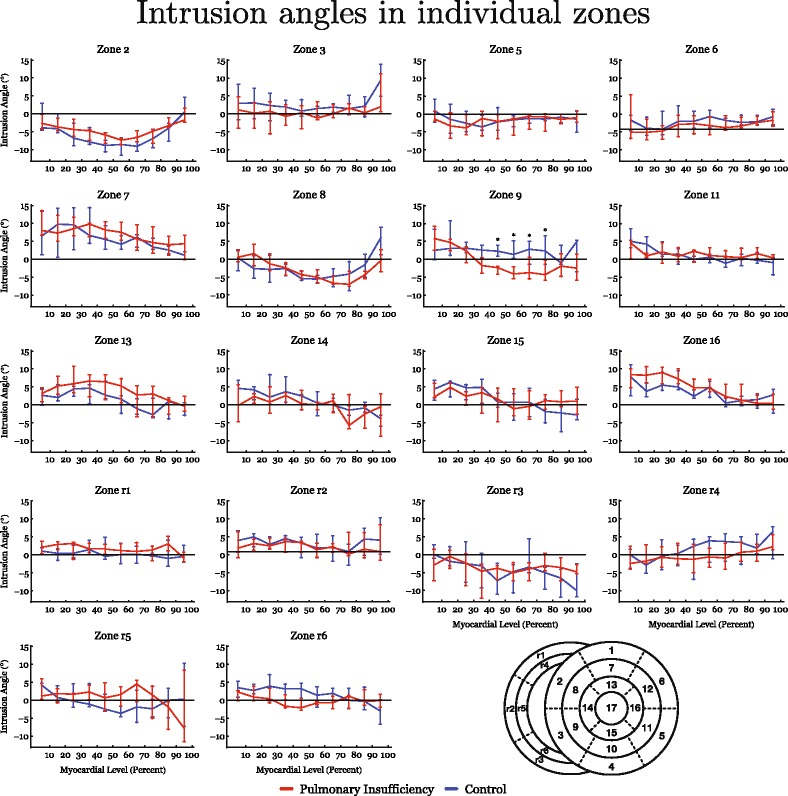
Detailed regional analysis based on data from individual zones in the entire heart. Transmural analyses of intrusion angles from sub-endocardium (0) to sub-epicardium (100). Asterisk indicates statistical significance in individual areas using Mann-Whitney U-test. In the down right corner a schematic representation of the zones is presented for reference

**Fig. 8:**
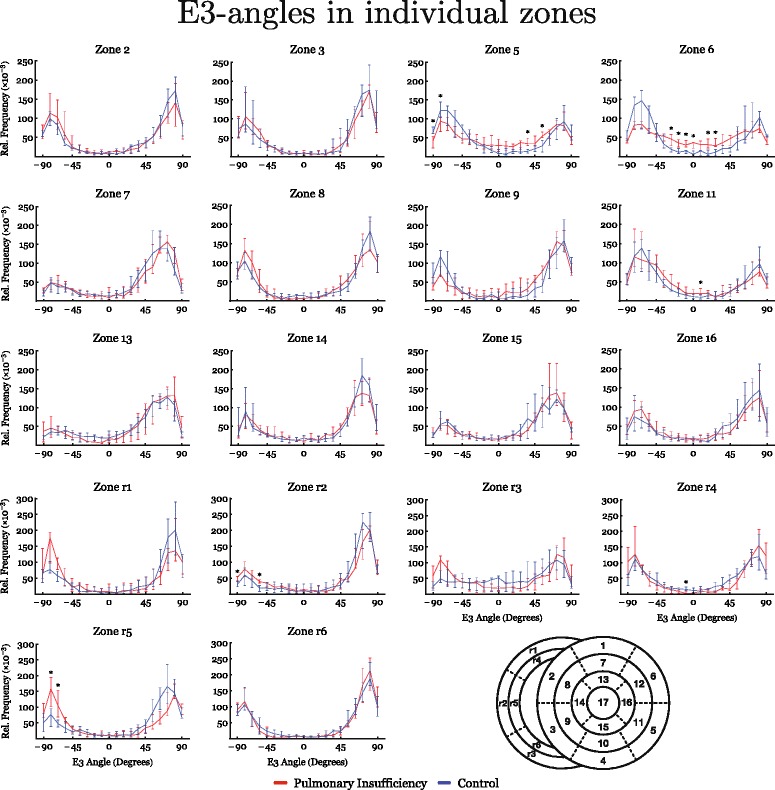
Regional analyses of E3-angles are shown in histograms. In the down right corner a schematic representation of the zones is presented for reference. Asterisk indicates statistical significance in individual areas using Mann-Whitney U-test

The results of diffusion tensor imaging analyses are shown in Figs. 3, 4, 5, 6, 7 and 8. Figure 3 shows histograms of the distributions of the helical, intrusional, and E3 angles while Fig. 4 shows the binned angles as a function of myocardial depth as originally proposed by Streeter and colleagues [8]. Detailed analyses of the individual zones can be found in Figs. 6, 7 and 8.

**Fig. 9:**
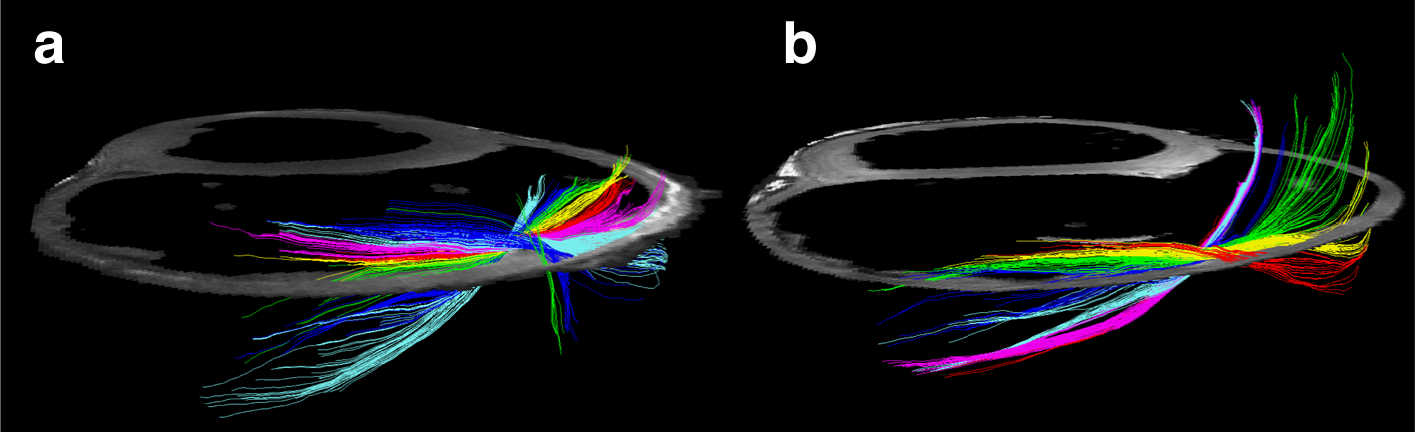
Tractography images of cardiomyocytes in the free wall of the right ventricle in controls (**a**) and right ventricular dilation (**b**). The tracks are limited to a length of 4 cm for the ease of interpretation. The colour code of the tracks does not represent any anatomical or physiological properties, but are added as a visual aid enabling the reader to distinguish between individual tracks. The helical angles of the tracts are approaching a more circumferential orientation in the dilated right ventricle (**b**)

As evidenced by both figures the helical angles differed mainly in the right ventricular walls, where in general a higher proportion of negative angles are present in the setting of pulmonary regurgitation. This is also evident from Fig. 9, where representative examples of tractographies from the animals with pulmonary regurgitation are compared with their controls. Minor differences were found in the left ventricular endocardium, while no differences were found in the interventricular septum. Statistical testing showed the differences for the right ventricle to be located predominantly in the ventricular midwall, where a decrease in helical angle of 11.5° was found (Table 2, *p* = 0.01).

**Table 2:** Helical Angulations of Cardiomyocytes

	Endocardium (0–33%)				Midwall (33–66%)				Epicardium (66–100%)			
	PI	Control	Diff.	*p*-value	PI	Control	Diff.	*p*-value	PI	Control	Diff.	*p*-value
LEFT VENTRICLE	31.0° (16.7;47.9)	37.7° (22.3;46.4)	−6.7°	NS	−5.8° (−16.3;-4.8)	−2.4° (−12.1;10.5)	−3.4°	NS	−35.7° (−42.3;-31.8)	−37.1° (−41.6;-29.3)	1.4°	NS
RIGHT VENTRICLE	23.2° (8.8;37.5)	33.3° (17.8;39.2)	−10.1°	NS	−3.1° (−6.7;6.6)	8.4° (−6.4;12.8)	−11.5°	0.01	−15.3° (−24.4;-1.3)	−13.3° (−21.4;−2.1)	-2°	NS
SEPTUM	36.4° (30;41.2)	33.6° (27;42.6)	2.7°	NS	−6.9° (−12.3;0.02)	−6.2° (12.3;-1.3)	−0.7°	NS	−41.2° (−51.7;-32.5)	−42.6° (−49.8;-37.7)	1.5°	NS
ENTIRE HEART	31.8° (18.5;41.4)	33.7° (22.5;43.4)	−1.9°	NS	−5.3° (−11.6;4.3)	−0.8° (−11.3;10.3)	−4.5°	0.01	−33.5° (−41.2;-19.5)	−32.2° (−41.6;-18.2)	−1.3°	NS

Angle analysis based on myocardial depth. Septal endocardium is the left ventricular endocardium while septal epicardium refers to the right ventricular endocardium. Data are shown as medians (interquartile range)

*NS* Statistically nonsignificant, *PI* Pulmonary insufficiency

The intrusional angles showed the most obvious changes in the right ventricle as seen in Fig. 3, where the proportion of surface parallel cardiomyocytes increased. Statistical testing on myocardial level only revealed significant differences in the septum by a decrease in intrusional angle in the right ventricular sub-endocardium of approximately 5° (*p* = 0.04), see Fig. 4.

Figure 3 also shows that significant differences for the E3 angles were found in both ventricles, with a slightly higher proportion of angles for the left ventricle with a value around zero, and conversely a lower proportion of angles with a numerically higher value. In the dilated right ventricle the proportion of E3 angles around −90° is more markedly increased, while the proportion around 90° is decreased. No differences were found for measurements taken in the ventricular septum. As shown in Fig. 5, E3 angles were distributed very heterogeneously within the myocardium. It was not justifiable, therefore, to plot them as a function of myocardial depth and hence the difference in angle distribution could not be attributed to a specific myocardial region. Because of this, we excluded E3 plots from Fig. 4.

## Discussion

**Fig. 10:**
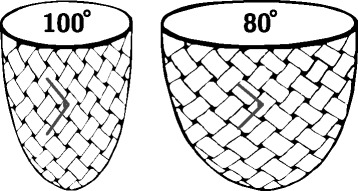
The wicker basket analogy. When the basket is dilated in the right panel the angle, between the wickers decreases from approximately 100° to 80°. In this basket the angle between the wickers decrease when the basket is dilated in exactly the same way as seen in the dilating right ventricle. The figure is not to be understood as a model of mural architecture and it is not to be interpreted as an image of how the myocardium is constructed. Contrary, the purpose of the figure is to illustrate the concept of what happens to the helical angles, depicted by the angles between the wickers, when the cavity dilates. No conclusions on E3 or intrusion angles can be made from this model

To the best of our knowledge, this is the first study to show that right ventricular dilation from pulmonary regurgitation leads to alterations in overall ventricular mural architecture. Significant remodelling was found in both ventricles, despite the fact that pulmonary regurgitation is often considered a strictly right ventricular pathology. We found that the helical ang​les ​of ​right ventricular cardiomyocytes approach a more circumferential orientation as a consequence of right ventricular dilation (Figs. 3, 4 and 9). We also observed an increase in the proportion of intrusional angles close to zero degrees in the right ventricle, along with an increase in the proportion of E3 angles around −90° as seen in the diastolic phase. The latter finding is as anticipated for the alterations of right ventricular helical and intrusional angles. We found a similar tendency in terms of helical angles measured in the left ventricle, but here the mural architecture was seemingly changed in the opposite direction in terms of the intrusional and E3 angles. The latter attributes assumed a more systole-like orientation. In the left ventricle, furthermore, the proportion of intrusional angles around zero degrees decreased in the setting of right ventricular dilation, while the E3 angles changed towards a more horizontal alignment, again as seen in the systolic heart [5]. Ferreira and associates suggested this conformation to be brought upon by failure of diastolic relaxation. Hyldebrandt and co-workers assessed the current model by conductance catheter technique [22]. They found an unaltered left ventricular isovolumetric relaxation constant (tau) whereas left ventricular compliance was decreased as judged by a significant increase in the end-diastolic pressure volume relationship. Hence left ventricular diastolic function is certainly impaired as is often seen in hypertrophy [28]. It is, therefore, very interesting that we were unable to show hypertrophy when comparing myocardial thickness between groups. This suggests that intramural myocardial remodeling precedes detectable transmural thickening. We found little if any change in the architecture of the septum, apart from a strictly localised decrease in the intrusional angles measured in the right ventricular sub-endocardium. Diffusion tensor imaging studies of the right ventricle are a rare sight in the published literature. We are aware of only three studies, of which our group has contributed two [6, 20, 29]. Our current study is, to the best of our knowledge, the first to explore the influence of right ventricular dilation on myocardial architecture. In our previous study of right ventricular hypertrophy produced by pulmonary banding [20], we found no differences between the hypertrophied hearts and their controls in terms of helical angles, but in that study we did not assess either intrusional angles or E3 angles. Our scan resolution at that time was also 64 times lower than in both our newer studies, which might mask smaller differences. As we have previously elucidated right ventricular pressure and volume overload indeed leads to two different manifestations of heart failure [22, 30]. In our most recent study, of right ventricular pressure overload​ in​persistent pulmonary hypertension in the newborn [6], ​we found alterations in myocyte angulations with both preserved cavitary volumes and with universal myocardial hypertrophy. This is the inverse pattern compared with our present study of right ventricular volume overload, in which we found that the hearts with dilation had increased their right ventricular volume without changing either of their ventricular mural thicknesses. At present, we have investigated the thin right ventricular wall with an appropriate imaging resolution comparable to that of other studies, and we have utilised an animal model that is anatomically and physiologically well documented [21, 22]. We have also achieved a degree of pulmonary regurgitation in our model comparable with that of earlier studies. Dilated cardiomyopathy in the left ventricle has been assessed with diffusion tensor imaging by several groups [7, 31, 32]. Li and associates, using genetically modified hamsters, were unable to show any changes in helical and transverse angles, but their specimens were scanned using only 6 diffusion encoding directions, and with a very large slice thickness [31]. The alignment of the myocytes, therefore, is averaged over quite large myocardial areas, despite the use of a reasonable in-plane resolution. The same is the case in a recent study in humans by Nielles-Vallespin and associates [7]. They were, however, able to detect a significant change in the orientation of the aggregated cardiomyocytes in spite of low resolution while the helical and transmural angles were unaltered. Like in our study the myocardium of left ventricular dilated cardiomyopathy had a configuration towards diastole. Contrary to this finding the hearts in the study of von Deuster and co-workers seems to be configured in a state resembling systole and, moreover, they detect an overall increase in the helical angle [32]. This difference can most likely be attributed to the difference in baseline characteristics between the studies and to the use of strain correction in the von Deuster study. Here the hearts are barely dilated, but merely hypertrophied as opposed to the hearts in the Nielles-Vallespin study, which are significantly morphologically dilated. In our study with higher resolution, we found changes in all angles measured. In the right ventricle in particular, the helical angles approached a more circumferential orientation. This finding supports the “wicker basket” analogy originally proposed by Streeter and colleagues in 1973 [33], as shown in our Fig. 10. On this basis, we presume that the change noted in helical angulation of the cardiomyocytes in the right ventricular wall is simply a mechanical consequence of ventricular dilation. The ideal response of the ventricle to acute volume overload is to enlarge. An increased end-diastolic volume then causes a right-shift on the Starling curve, leading to altered working conditions for the individual cardiomyocytes. When this is normalised by secondary hypertrophy, a larger, but morphologically normal, right ventricle should cope with these alterations quite easily. A previously normal right ventricle, however, cannot expand isometrically while maintaining its normal shape, mainly because it is fixed to the left ventricle, which does not change its size. The “wicker basket” analogy shows that, if the chamber were allowed to lengthen as much as it widens, then the angles would not be disturbed (Fig. 10). In contrast, if the ventricle, as in this illustration, is obliged to undergo allometric scaling, the normal balance between the inner and outer helical angles will be disturbed. This can be dysfunctional, as it will lead to a mismatch between the contraction capabilities of the individual cardiomyocytes and the need for myocardial deformation required to maintain stroke volume [34]. As observed, the result will be a change in the architecture of the myocardial mesh. The dilation is effected mainly by changes in ventricular short-axis dimensions other than its length, and the myocardium is not capable of reforming itself in the timescale provided. Hence, the inevitable consequence of transverse dilation of the ventricle is that the aggregated cardiomyocytes will become more surface parallel in terms of decreased intrusional angles, with their helical angles flattened towards zero. This will produce an inevitable compromise between the normal balance between the helical angles of the inner and outer zones. An increased end-diastolic volume causes a right-shift on the Starling curve, leading to altered working conditions for the individual cardiomyocytes. To accommodate these changes, remodelling to some extent on a cellular level must also take place. We found right ventricular dilation with no thinning of the ventricular wall, meaning that the myocardium must have increased its mass. This augmentation of myocardial mass must be brought upon by cellular hypertrophy, because the number of cells in the myocardium cannot increase in number, at least not that much and that quick. Myocardial hypertrophy entails an increase in cell thickness, or cell length, or both. In this case the cells must mainly increase in length because the original wall thickness is maintained, but the radius of the ventricle is enlarged. Hypertrophy, however, does not allow any realignment of the myocyte orientation and hence normalization of the myocardial contractional forces. As described we have attempted to maintain the shape of the ventricles during fixation and we were unable to detect any difference in wall thickness. It is, however, impossible to reproduce the same loading conditions that apply in-vivo when imaging of the heart ex-vivo. In particular, when imaging the thin walled right ventricular myocardium, loading conditions could have a major impact. It is, therefore, not given that in-vivo imaging of the right ventricle under true loading conditions would produce the same results. This is an important limitation to this study. As previously discussed in-vivo imaging of the right ventricle is not feasible with sufficient resolution given the present developmental state of the technology. It has, however, been suggested that in-vivo diffusion tensor imaging of the interventricular septum can provide information on right ventricular function [35], but conclusions on its morphology and remodelling cannot be made from investigation of the interventricular septum. In this study we have analysed the changes in myocyte orientation in formalin fixed hearts. Given the well-known effects of formalin on tissues [36] it is reasonable to speculate that the process of fixation could skew our results. It is, however, well-documented that myocyte orientations as assessed with histology are comparable with those calculated from diffusion tensor imaging in both fresh [37] and perfusion fixed hearts [38]. We have, moreover, recently documented that the diffusion properties of cardiac tissue are comparable between fresh and perfusion fixed cardiac tissue [39].

We found an increase in the amount of circumferentially oriented cardiomyocytes in the right ventricle as was also found in the study of Sanchez-Quintana and co-workers achieved by dissecting human hearts with tetralogy of Fallot [9]. The physiological mechanisms of the myocardial remodelling observed in the presented model and in tetralogy of Fallot, however, are quite different. In this study, we have investigated a volume overload model, whereas tetralogy of Fallot is a pressure overload disease. Although the proportion of circumferentially oriented cardiomyocytes increases in the dilation model, the etiology is quite different from that observed in the setting of tetralogy. It could be argued that the increase of circumferentially oriented myocytes in tetralogy is a compensatory mechanism as part of myocardial hypertrophy, whereas in dilation the increase in circumferential myocytes is merely a disadvantageous mechanical consequence of expanding the cavity of the ventricle. Realignment of the cardiomyocytes must also lead to alterations in how the contraction of the myocytes affects the ventricle. Mathematical models as presented by Sallin and associates and ourselves have argued that the presence of a helical angle is mandatory in order to produce an ejection fraction within physiologically normal range [40, 41]. Moreover, a recent study in humans with situs inversus totalis by Khalique and co-workers found an altered helical angle pattern in the left ventricle leading to reduced torsion [42]. Even though all three of these works only study the left ventricle, it is highly likely that the myocardial rearrangement in the dilated right ventricle towards a helical angle of zero degrees is not beneficial for right ventricular cardiodynamics. It could very well be part of the explanation of the heart failure that will eventually result from ventricular dilation [22].

Our study has also shown that left ventricular myocardial remodelling is brought about by pulmonary regurgitation, as evidenced by the observed changes in the distribution of the E3 angles. The proportion of angles with a numerically low value around zero increases concomitant with right ventricular dilation. This phenomenon has been described previously, since it is the same pattern as seen when the heart approaches the systolic contractional state [5]. The systolic-like configuration of the E3 angles in the present study, however, is not associated with the anticipated mural thickening. The helical angles in the left, and especially the right, ventricle furthermore have a more diastolic configuration, with values closer to zero. Hence, there is a mismatch between the state of contraction and the configuration of the cardiomyocytes. This is in keeping with our findings in our sheep model of right ventricular pressure overload in persistent pulmonary hypertension of the newborn [6]. In this setting, we found the reverse situation, with the cardiomyocytes configured in a more diastolic state in spite of myocardial hypertrophy. In both studies, therefore, we describe types of mismatch between contraction state and myocyte architecture that could potentially aid in the explanation of heart failure in myocardial remodelling. There is great difference in literature on how to quantify the orientation of aggregations of cardiomyocytes. Some quantify them as absolute values [7], while others, such as ourselves consider the orientations with a sign. Our results underline why this is important. When contemplating Fig. 8 it is clear that positive and negative E3-angles are not equally distributed and, moreover, changes in the distributions caused by right ventricular dilation do not affect positive and negative angles equally. Differentiation between positive and negative angles is, therefore, indeed important although the functional interpretation of the differences between positive and negative E3 angles is far from clarified. In addition, we cannot unequivocally answer the question as to why right ventricular volume overload, in time, causes heart failure. Heart failure is well recognised to be a multifaceted disease, with a complex aetiology which probably also varies depending on the cause of failure [43]. We have now seen several cases of myocardial remodelling in the development of heart failure, but questions regarding the threshold relative to clinical heart failure, and the reversibility of the myocardial remodelling, have never been investigated. If this proved possible, then the specific role of remodelling in heart failure could potentially be more clearly elucidated.

## Conclusion

Our present study indicates that remodelling as seen relative to mural architecture may play a part in the pathophysiology of heart failure in right ventricular dilation. This remodelling may simply come to pass by mechanical effects on the myocardium brought upon by pulmonary regurgitation. A mismatch is found between the alignment of the aggregated cardiomyocytes and the cardiac contractional state indicating that remodelling might not fully achieve the benefit intended.
